# Effects of Dietary Methionine and Lysine Balance on Intestinal Function and Immune Organ Development in Wanxi White Geese During the Brooding Period

**DOI:** 10.3390/ani16060872

**Published:** 2026-03-11

**Authors:** Shaoqi Shi, Changsheng Jiang, Suting Fang, Leilei Li, Xiaojin Li, Ahmed H. Ghonaim, Man Ren, Shenghe Li

**Affiliations:** 1 Anhui Provincial Key Laboratory of Animal Nutritional Regulation and Health, College of Animal Science, Anhui Science and Technology University, Chuzhou 233100, China; 18255049355@163.com (S.S.); jiangcs@ahstu.edu.cn (C.J.); 15255691160@163.com (S.F.); leill3@163.com (L.L.); lixj@ahstu.edu.cn (X.L.); 2 Anhui Engineering Technology Research Center of Pork Quality Control and Enhance, Anhui Science and Technology University, Chuzhou 233100, China; 3 National Key Laboratory of Agricultural Microbiology, College of Animal Sciences and Veterinary Medicine, Huazhong Agricultural University, Wuhan 430070, China; a.ghonaim@webmail.hzau.edu.cn; 4 Desert Research Center, Cairo 11435, Egypt

**Keywords:** Wanxi white geese, methionine, lysine, protein level, intestinal function, immune function

## Abstract

(1) Background: This experiment investigated the effects of balancing methionine or lysine under different dietary protein levels on intestinal function and immune organ development in Wanxi white geese during the brooding period. (2) Methods: A total of 180 one-day-old Wanxi white geese were randomly divided into six groups using a 2 × 3 factorial design, with amino acid balance (lysine or methionine) and dietary crude protein levels (20%, 18%, and 16%) as experimental factors. Each group consisted of five replicates with six geese per replicate. The feeding trial lasted 28 days. (3) Results: The lysine-balanced diet significantly increased jejunal lipase activity in 14-day-old geese (*p* < 0.05). In contrast, the methionine-balanced diet significantly enhanced ileal trypsin, maltase, and amylase activities at 14 days, as well as ileal trypsin activity at 28 days (*p* < 0.05). Lysine balance markedly improved jejunal weight and muscularis thickness, ileal weight, villus height, and villus-to-crypt (V/C) ratio in 14-day-old geese, and further increased jejunal villus height and ileal crypt depth and muscularis thickness at 28 days (*p* < 0.05). Methionine balance significantly elevated total antioxidant capacity (T-AOC) in the jejunum and ileum of geese at both 14 and 28 days (*p* < 0.05). Moreover, the methionine-balanced group exhibited a significantly higher thymus index and spleen index in 28-day-old geese compared with the lysine-balanced group (*p* < 0.05). Notably, geese fed the 18% crude protein diet showed significantly greater follicular and medullary areas of the bursa of Fabricius at 14 days than those fed 20% or 16% protein diets (*p* < 0.05). (4) Conclusions: Appropriate dietary protein reduction combined with balanced lysine or methionine supplementation effectively enhances intestinal digestive function, antioxidant capacity, and immune organ development in Wanxi white geese. Among the tested treatments, a dietary protein level of 18% produced the most favorable overall outcomes during the brooding period.

## Simple Summary

Protein is one of the most expensive components of poultry feed and reducing dietary protein while maintaining animal health is an important goal in goose production. This study investigated whether balancing two essential amino acids, lysine or methionine, could improve intestinal function and immune development in Wanxi white geese fed diets with different protein levels during the brooding period. The results showed that appropriate lysine or methionine supplementation enhanced digestive enzyme activity, intestinal structure, antioxidant capacity, and immune organ development. In particular, a diet containing 18% crude protein produced the most favorable overall effects. These findings suggest that moderate protein reduction combined with balanced amino acid supplementation can support intestinal health and immune function while potentially reducing feed costs in Wanxi white geese.

## 1. Introduction

Amino acids are the fundamental building blocks of proteins. Deficiency of any essential amino acid in animal feed can impair protein synthesis, thereby adversely affecting growth, development, and overall health [1,2,3,4,5,6]. Lysine is typically the first limiting amino acid in the diets of most mammals; therefore, the ideal amino acid profile in livestock and poultry feed is commonly expressed relative to lysine [7]. From the 1950s to the 1990s, Ren et al. [8] and Baker et al. [9] proposed the concept of ideal protein for poultry and swine diets, while Cole and Fuller [10] suggested that pig diets should provide an optimal ratio of essential amino acids using lysine as the reference amino acid. When amino acids with similar chemical structures or metabolic properties are supplied in imbalanced proportions, amino acid antagonism may occur, leading to reduced feed intake, abnormal behavior, and impaired growth and development [11].

Previous studies have demonstrated that the ideal protein pattern in feed does not always accurately reflect the amino acid composition required by the animal’s body [12]. Individual dietary amino acids differ in their rates of digestion, absorption, and catabolism in the small intestine, and plasma amino acids exert distinct metabolic functions in different tissues [13]. Although recommended amino acid requirement tables are available for different species, growth stages, and production systems, actual amino acid requirements may vary due to changes in diet composition, genetic selection, and management practices [9,12]. Proper balancing of dietary amino acids can enhance growth rate, production performance, immune function, and disease resistance, ultimately improving animal productivity and health [10,14]. Therefore, careful consideration of essential amino acid balance in feed formulation is critical to meeting animals’ comprehensive amino acid nutritional requirements [13,15].

As key nutritional components in poultry diets, dietary protein and its constituent amino acids play a pivotal role in promoting animal growth, improving feed conversion efficiency, and enhancing immune function [16]. However, despite the central importance of protein in animal nutrition, substantial gaps remain in understanding the precise regulation of amino acid balance and its effects on the growth and development of poultry populations [17]. In poultry production, lysine and methionine are generally considered the most limiting amino acids, as their deficiency restricts protein synthesis efficiency and negatively affects growth and health [18,19].

While the amino acid requirements for broilers have been extensively studied, research on the specific amino acid requirements of geese remains relatively limited. Previous studies on geese have primarily focused on the basic requirements of crude protein and sulfur-containing amino acids. Min et al. [20] reported that the optimal dietary protein level for goslings aged 0–28 days ranges from 17% to 19%, while a diet containing 16% protein with balanced lysine significantly promoted weight gain during the first 14 days of age. Ashour et al. [15] demonstrated that a diet containing 16% protein supplemented with 0.65% methionine and cystine significantly improved growth performance, carcass traits, and meat composition in Egyptian geese aged 12–24 weeks. Alagawany et al. [21] found that a diet with 16% protein level and 2800 kcal/kg metabolizable energy maintained growth performance and physiological indicators, including vitality, kidney function, antioxidant capacity, immune status, and blood lipid profiles, in Egyptian geese aged 1–12 weeks. Xi et al. [22] reported that a diet containing 18% protein and 5% crude fiber was more conducive to maintaining intestinal microbial balance and preventing gout in Taizhou geese aged 1–21 days. Yang et al. [23] observed that increasing dietary methionine concentration linearly enhanced body weight and average daily gain in goslings. Recent studies have also indicated that appropriate adjustment of dietary protein levels and amino acid ratios not only optimizes growth performance but also improves metabolic efficiency and reduces environmental pollution, such as nitrogen emissions [24,25,26,27,28]. However, systematic investigations on the effects of low-protein diets with different amino acid balances in Wanxi white geese remain limited.

Wanxi white goose is a well-recognized medium-sized goose breed in China, valued for its large body size, high-quality meat, superior down quality, and strong disease resistance [29]. However, this breed exhibits seasonal laying behavior and relies heavily on natural hatching. Consequently, Wanxi white geese have a short laying period, prolonged brooding behavior, low natural reproductive performance, and strong broodiness, resulting in annual egg production of fewer than 30 eggs and relatively low immunity. Therefore, the present study aimed to investigate the effects of balancing methionine or lysine under low-protein diets on intestinal health and immune function of Wanxi white geese. By evaluating different protein levels and amino acid supplementation strategies, this study seeks to provide a theoretical basis for optimizing feed formulations and improving production performance and immune capacity in Wanxi white geese.

## 2. Materials and Methods

### 2.1. Animal Ethics Statement

All animal procedures were reviewed and approved by the Animal Ethics Committee of Anhui Science and Technology University (protocol number 2023224) and conducted in accordance with the “Guidelines for the Care and Use of Test Animals” of Anhui Province.

### 2.2. Experimental Design and Feeding Management

Wanxi white geese were obtained from the Lu’an Junming Wanxi White Goose Improved Breeding Base (Lu’an, China). A total of 180 one-day-old Wanxi white geese (equal numbers of males and females) were randomly allocated into six treatment groups, each comprising five replicates with six birds per replicate. The experimental period lasted 28 days. The rearing temperature was maintained at 30 °C for the first week and gradually decreased by 2 °C per week until it reached room temperature. The photoperiod was regulated dynamically according to the age of the goslings: 23 h of light and 1 h of darkness during the first week, 18 h of light and 6 h of darkness during the second week, 16 h of light and 8 h of darkness during the third week, and 12 h of light and 12 h of darkness during the fourth week. The stocking density was maintained at 6 birds/m^2^, providing sufficient space for ad libitum access to feed and water.

A 2 × 3 factorial experimental design was employed, with amino acid type (lysine or methionine) and dietary crude protein (CP) level as the two main factors. The CP levels were set at 20% (high), 18% (medium), and 16% (low). The experimental treatments were as follows: Group A (20% CP + 1% lysine); Group B (18% CP + 1% lysine); Group C (16% CP + 1% lysine); Group D (20% CP + 0.5% methionine); Group E (18% CP + 0.5% methionine); Group F (16% CP + 0.5% methionine). The average initial body weights of the goslings for groups A, B, C, D, E, and F were 87.14 g, 87.57 g, 88.61 g, 88.59 g, 88.59 g, and 89.83 g, respectively.

The experimental design is presented in Table 1, and the ingredient composition and nutrient levels of the diets are shown in Table 2.

The geese were reared in a floor-rearing system with plastic netting under natural lighting conditions and had ad libitum access to feed and water. The rearing environment was maintained in a clean, well-ventilated condition, and routine management practices were implemented consistently across all groups. Vaccination and health management were conducted strictly according to the standard immunization program.

### 2.3. Sample Collection

On days 14 and 28 of the experiment, geese were slaughtered for sample collection. Five goslings with body weights close to the average of their respective replicates were randomly selected from each group. The birds were fasted for 12 h with ad libitum access to water and then euthanized. After slaughter, the jejunum, ileum, cecum, thymus, spleen, and bursa of Fabricius were excised. For histomorphological analysis, tissue samples (approximately 2 cm in length) were collected from the midpoint of the jejunum (between the entry of the bile ducts and Meckel’s diverticulum) and the distal ileum (approximately 2 cm proximal to the ileocecal junction). These samples were gently flushed with physiological saline to remove contents and immediately fixed in 4% paraformaldehyde.

Intestinal contents were gently flushed with physiological saline, and tissue samples were fixed in 4% paraformaldehyde for histomorphological analysis. The mucosa of the jejunum and ileum was carefully scraped using sterile glass slides and stored at −80 °C for the determination of digestive enzyme activities. In addition, sections of the mid-jejunum and terminal ileum were immediately snap-frozen in liquid nitrogen and stored at −80 °C for antioxidant analysis.

### 2.4. Determination of Intestinal Digestive Enzyme Activities

Digestive enzyme activities in the jejunal and ileal mucosa of 14-day-old and 28-day-old Wanxi white geese were determined using commercially available assay kits according to the manufacturers’ instructions. The details of the kits used are provided in Table 3.

### 2.5. Determination of Intestinal Relative Length and Organ Index

The thymus, spleen, bursa of Fabricius were trimmed of surrounding fat and mesentery and weighed. The natural lengths of the jejunum, ileum, and cecum were measured using a flexible tape measure.

Intestinal relative length (cm/g) = intestinal length (cm)/body weight (g)

Organ index = organ weight (g)/live body weight before slaughter (g) × 100%

### 2.6. Organs Morphology

Tissues fixed in 4% paraformaldehyde were dehydrated, embedded in paraffin, sectioned, and stained with hematoxylin and eosin (H&E). Histological observations were performed under a light microscope, and morphometric measurements were conducted using Image-Pro Plus 6.0 software.

### 2.7. Determination of Antioxidant Indicators

The activities of reduced glutathione (GSH), total antioxidant capacity (T-AOC), catalase (CAT), and malondialdehyde (MDA) levels in jejunal and ileal mucosa were determined using commercial assay kits following the manufacturers’ protocols. The details of the antioxidant kits are listed in Table 4.

### 2.8. Statistical Analysis

All data were analyzed using SPSS statistical software (version 25.0). Two-way analysis of variance (ANOVA) was performed to evaluate the main effects of dietary protein levels (20%, 18%, and 16%), amino acid balance mode (lysine or methionine), and their interactions. Differences among means were assessed using the least significant difference (LSD) test. Values within the same column with identical or no superscript letters indicate no significant difference (*p* > 0.05), different lowercase letters indicate a significant difference (*p* < 0.05), and different uppercase letters indicate an extremely significant difference (*p* < 0.01).

## 3. Results

### 3.1. Effects of Lysine- and Methionine-Balanced Diets on Digestive Enzyme Activities

As shown in Table 5, in 14-day-old Wanxi white geese, dietary amino acid balance significantly affected intestinal digestive enzyme activities. Jejunal lipase activity (*p* < 0.05) of the lysine-balanced group was significantly higher in the lysine-balanced group than that of the methionine-balanced group (*p* < 0.05). In contrast, the methionine-balanced diet significantly increased ileal trypsin (*p* < 0.05), maltase (*p* < 0.01), and amylase (*p* < 0.01) activities compared with the lysine-balanced diet.

Dietary protein level also influenced enzyme activities. Compared with the 18% and 20% CP diets, the 16% CP diet markedly increased jejunal trypsin activity (*p* < 0.01). A significant interaction between amino acid balance and protein level was observed for ileal trypsin activity (*p* < 0.01), indicating that the response of trypsin activity depended on the combined dietary treatments.

As shown in Table 6, in 28-day-old geese, ileal trypsin activity remained significantly higher in the methionine-balanced group than in the lysine-balanced group (*p* < 0.05). In addition, the 20% and 18% CP diets significantly increased jejunal lipase activity compared with the 16% CP diet (*p* < 0.05). The highest ileal trypsin activity was observed in the 20% CP group, which was significantly greater than that in the 18% and 16% CP groups (*p* < 0.05).

### 3.2. Effects of Lysine- and Methionine-Balanced Diets on Intestinal Relative Length and Weight

As presented in Table 7, dietary protein level significantly affected jejunal relative length in 14-day-old geese, with the 18% CP diet producing a longer jejunum compared with the 16% CP diet (*p* < 0.05). Neither amino acid balance nor protein level significantly influenced the relative length of the ileum or cecum at 14 or 28 days of age (*p* > 0.05). However, a significant amino acid × protein interaction was detected for jejunal relative length in 14-day-old geese (*p* < 0.05).

As shown in Table 8, the lysine-balanced diet significantly increased the relative weights of the jejunum, ileum, and cecum in 14-day-old geese compared with the methionine-balanced diet (*p* < 0.05). No significant effects of protein level or amino acid × protein interaction were observed on relative intestinal weight at either age.

### 3.3. Effects of Lysine- and Methionine-Balanced Diets on Intestinal Morphology

**Figure 1:**
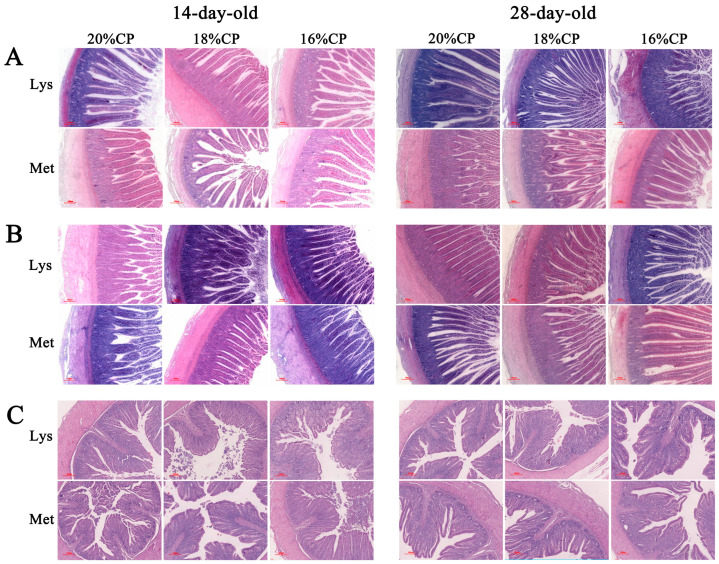
The morphology of the jejunum (**A**), ileum (**B**), and cecum (**C**) of Wanxi white geese at 14-day-old and 28-day-old. Scale bars are 100 µm.

As shown in Table 9, in 14-day-old geese, the lysine-balanced diet significantly increased jejunal muscularis thickness compared with the methionine-balanced diet (*p* < 0.01). Compared with the 18% CP diet, the 16% CP diet significantly increased villus height and crypt depth, while significantly reducing muscularis thickness (*p* < 0.01 or *p* < 0.05). In 28-day-old geese, the lysine-balanced diet significantly increased jejunal villus height (*p* < 0.01). The 16% CP diet significantly reduced villus height and muscularis thickness compared with the 18% CP diet (*p* < 0.01 or *p* < 0.05) (Figure 1A).

As shown in Table 10, the lysine-balanced diet significantly increased ileal villus height and villus-to-crypt (V/C) ratio (*p* < 0.01), while significantly reducing muscularis thickness (*p* < 0.01). In contrast, in 28-day-old geese, the lysine-balanced diet significantly increased crypt depth and muscularis thickness (*p* < 0.05 or *p* < 0.01) but reduced the V/C ratio (*p* < 0.05). Regarding protein level, the 16% CP diet significantly increased ileal muscularis thickness in 14-day-old geese (*p* < 0.01). In 28-day-old geese, the 16% CP diet significantly increased villus height (*p* < 0.05) and decreased muscularis thickness (*p* < 0.01) compared with the 18% CP diet (Figure 1B).

As shown in Table 11, in 28-day-old geese, the lysine-balanced diet significantly reduced cecal villus height compared with the methionine-balanced diet (*p* < 0.05). In 14-day-old geese, the 18% CP diet significantly increased villus height and V/C ratio compared with the 20% CP diet (*p* < 0.05). In 28-day-old geese, the 18% CP diet significantly reduced crypt depth while increasing the V/C ratio compared with the 20% CP diet (*p* < 0.05) (Figure 1C).

Overall, a lysine-balanced diet promoted intestinal morphological development, particularly in the jejunum and ileum of Wanxi white geese.

### 3.4. Effects of Lysine- and Methionine-Balanced Diets on Intestinal Antioxidant Capacity

As shown in Table 12, compared with the lysine-balanced group, the methionine-balanced diet significantly increased jejunal total antioxidant capacity (T-AOC) in both 14- and 28-day-old geese (*p* < 0.01 or *p* < 0.05). In addition, the 16% and 18% CP diets significantly increased jejunal T-AOC in 14-day-old geese (*p* < 0.05). In 28-day-old geese, these diets significantly increased malondialdehyde (MDA) content and catalase (CAT) activity (*p* < 0.05).

As shown in Table 13, in the ileum, the methionine-balanced diet significantly increased T-AOC in 14-day-old geese (*p* < 0.05). In 28-day-old geese, the methionine-balanced diet further increased T-AOC (*p* < 0.01) and significantly reduced MDA content (*p* < 0.01). The 16% CP diet significantly increased ileal MDA content and CAT activity in 14-day-old geese compared with the 20% CP diet (*p* < 0.05).

As shown in Table 14, the methionine-balanced diet significantly increased GSH and CAT activities in the cecum of 14-day-old geese (*p* < 0.05), whereas dietary protein level had no significant effect on cecal antioxidant indices (*p* > 0.05).

Collectively, these results indicate that a methionine-balanced diet enhances intestinal antioxidant capacity in Wanxi white geese.

### 3.5. Effects of Lysine- and Methionine-Balanced Diets on Thymus Development

As shown in Table 15, in 14-day-old geese, thymus weight and thymus index were not significantly affected by amino acid balance or protein level (*p* > 0.05). In 28-day-old geese, the thymus index was significantly higher in the methionine-balanced group than in the lysine-balanced group (*p* < 0.05).

**Figure 2:**
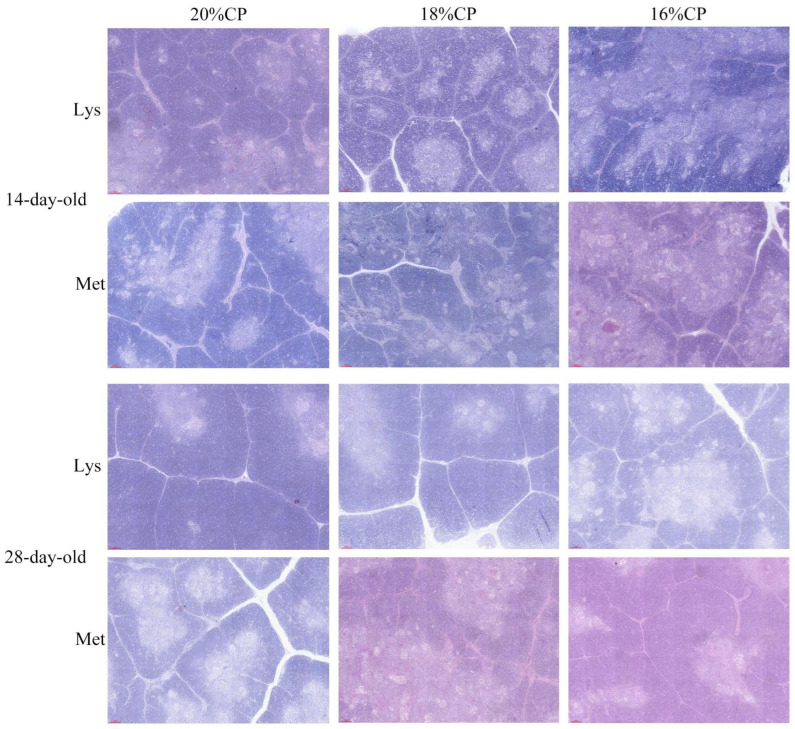
The thymus tissue structure of Wanxi white goose. Scale bars are 100 µm.

Histological observations showed that the 18% CP lysine-balanced group exhibited thicker thymic capsules, clearer septa, and well-defined thymic lobules in 14-day-old geese. In 28-day-old geese, thymic lobules increased in both size and number, with the 18% CP lysine-balanced group showing the most intact morphology (Figure 2).

As shown in Table 16, no significant effects of amino acid balance, protein level, or their interaction were observed on the relative cortical area, medullary area, or cortex-to-medulla ratio at either age (*p* > 0.05).

### 3.6. Effects of Lysine- and Methionine-Balanced Diets on Bursa of Fabricius Development

As shown in Table 17, the bursa index was not significantly affected by dietary treatments in 14-day-old geese (*p* > 0.05). In 28-day-old geese, the methionine-balanced diet significantly increased the bursa index compared with the lysine-balanced diet (*p* < 0.05).

**Figure 3:**
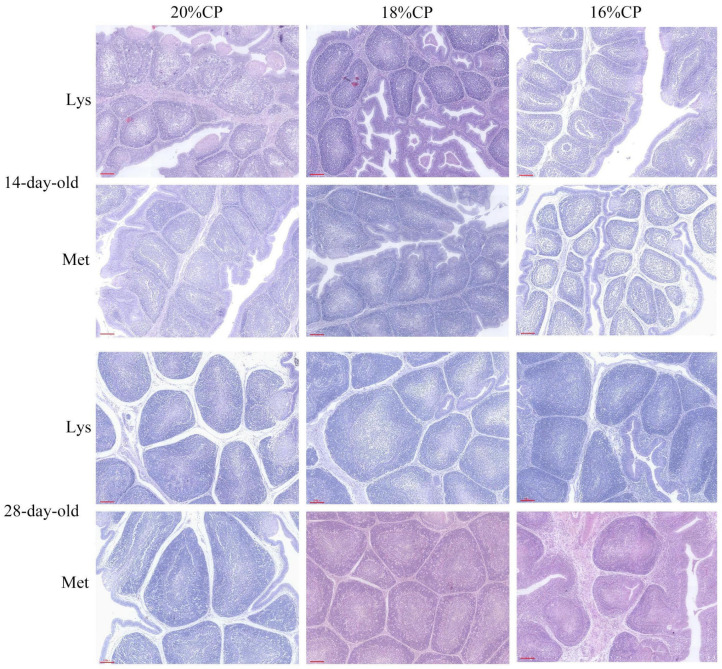
The bursa of Fabricius tissue structure of Wanxi white goose. Scale bars are 100 µm.

Histological examination revealed increased follicle number and area with age (Figure 3). As shown in Table 18, in 14-day-old geese, the 18% CP diet significantly increased follicle and medullary areas compared with the 20% and 16% CP diets (*p* < 0.01). In 28-day-old geese, follicle area was significantly greater in the 20% and 18% CP diets than in the 16% CP diet (*p* < 0.05). No significant amino acid × protein interactions were detected.

### 3.7. Effects of Lysine- and Methionine-Balanced Diets on Spleen Development

As shown in Table 19, spleen index was not significantly affected by dietary treatments in 14-day-old geese (*p* > 0.05). In 28-day-old geese, the methionine-balanced diet significantly increased the spleen index compared with the lysine-balanced diet (*p* < 0.05).

**Figure 4:**
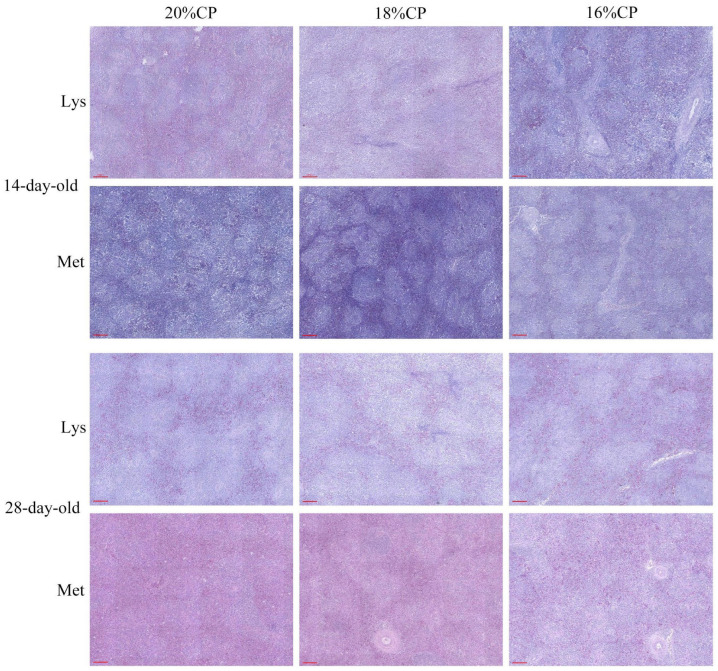
The spleen tissue structure of Wanxi white goose. Scale bars are 100 µm.

Histological analysis showed normal splenic architecture at both ages, with clear boundaries between red and white pulp and no pathological alterations. Neither amino acid balance nor protein level significantly affected spleen morphology (*p* > 0.05) (Figure 4).

## 4. Discussion

Gastrointestinal digestive enzyme activity is a key indicator of digestive efficiency and metabolic capacity in poultry [29,30]. Dietary protein and amino acids not only provide substrates for digestive enzyme synthesis but also regulate enzyme secretion through neuroendocrine mechanisms [31]. In the present study, a dietary crude protein (CP) level of 20% significantly increased ileal trypsin activity in 28-day-old Wanxi white geese, whereas the 18% CP diet enhanced jejunal lipase activity at the same age. In contrast, the 16% CP diet increased jejunal trypsin activity in 14-day-old geese but reduced jejunal lipase activity at 28 days of age. These findings suggest that digestive enzyme responses to dietary protein levels are both age- and intestinal segment-specific. The finding that the 18% CP diet yielded the most favorable outcomes suggests a trade-off between nutritional sufficiency and metabolic efficiency. While the 20% CP diet provided abundant nitrogen, the excess protein deamination may increase metabolic burden and oxidative stress, potentially offsetting growth benefits. Conversely, the 16% CP diet, despite amino acid balancing, might not provide a sufficient non-essential nitrogen pool or other limiting amino acids to support maximal intestinal development and enzyme synthesis. The 18% CP level, when balanced with limiting amino acids, appears to offer an optimal ‘sweet spot’ that sustains high digestive enzyme activity (e.g., jejunal lipase) and robust immune organ development (e.g., bursa of Fabricius follicles) without incurring the physiological costs associated with processing excess dietary protein.

Previous studies have shown that excessively high or low dietary protein levels may stimulate compensatory increases in jejunal trypsin and lipase activities in pigs and poultry, potentially due to intestinal irritation or non-dietary factors such as water intake. However, Bikker et al. [32] reported that dietary protein levels did not significantly affect small intestinal disaccharidase or aminopeptidase activities in early-weaned piglets, indicating species- and developmental-stage differences. In the current study, balanced lysine supplementation significantly increased jejunal lipase activity in 14-day-old geese, whereas balanced methionine supplementation enhanced trypsin, maltase, and amylase activities in the ileum at 14 days and increased ileal trypsin activity at 28 days. These results indicate that targeted amino acid supplementation can partially compensate for reduced dietary protein by supporting endogenous digestive enzyme activity.

Reducing dietary CP inevitably decreases overall nitrogen and amino acid intake, which can limit de novo protein synthesis and downregulate the mRNA translation of pancreatic and intestinal digestive enzymes [30,33]. However, essential amino acids are not only building blocks for these enzymes but also crucial regulators of overall gut function [14]. Specifically, an adequate supply of limiting amino acids, such as lysine and methionine, ensures sufficient substrate availability for the continuous synthesis and secretion of digestive enzymes in the gastrointestinal tract. Previous studies have demonstrated that optimizing the amino acid profile in low-protein diets effectively restores pancreatic enzyme secretion and intestinal absorptive capacity in poultry [10,34]. Therefore, the enhanced lipase and trypsin activities observed in the 18% CP group supplemented with balanced Lys or Met are likely attributable to the restoration of the amino acid pool necessary for optimal enzyme expression and synthesis, rather than merely a compensatory stress response.

The significant interactions observed between protein levels and amino acid sources, particularly for ileal trypsin activity and jejunal morphology, highlight the compensatory role of specific amino acids under protein-restricted conditions. Our results showed that methionine supplementation significantly enhanced trypsin activity specifically at lower protein levels (16% and 18%), but not at the high protein level (20%). This suggests that when dietary protein is insufficient to drive spontaneous enzyme synthesis, methionine may act as a critical signaling molecule or substrate to upregulate pancreatic enzyme secretion, thereby improving protein digestibility. This interaction underscores the necessity of precise amino acid balancing when formulating low-protein diets to prevent compromised digestive function.

Intestinal morphology, including length, weight, and structural characteristics, directly influences digesta retention time and nutrient absorption efficiency [35]. In this experiment, the 18% CP diet significantly increased the relative jejunal length of 14-day-old geese. Additionally, balanced lysine supplementation significantly increased the relative weights of the jejunum, ileum, and cecum at 14 days of age. These findings suggest that lysine plays a particularly important role in intestinal development during the brooding period and may indirectly contribute to enhanced growth performance by promoting intestinal maturation.

The jejunum and ileum are the primary sites of nutrient absorption in poultry, and villus height, crypt depth, and villus-to-crypt (V/C) ratio are widely used indicators of intestinal health and functional capacity [36]. Degradation of lysine and other basic amino acids in the small intestine has been shown to regulate villus architecture, crypt morphology, and mucosal thickness [37]. In the present study, balanced lysine supplementation significantly increased jejunal muscle layer thickness, ileal villus height, and V/C ratio in 14-day-old geese, as well as jejunal villus height, ileal crypt depth, and muscle layer thickness at 28 days. Balanced methionine supplementation increased ileal muscle layer thickness at 14 days and the V/C ratio at 28 days.

Methionine has been reported to play a critical role in maintaining intestinal barrier integrity, and in ovo methionine administration can enhance intestinal development during embryogenesis [38]. Consistent with these findings, our results demonstrate that both lysine and methionine exert significant regulatory effects on intestinal tissue morphology. Moreover, low-protein diets have been shown to increase villus height and reduce crypt depth, thereby enlarging the absorptive surface area and improving digestive efficiency [39,40]. In the present study, dietary protein level significantly influenced intestinal morphology in an age- and segment-dependent manner. Significant interactions between protein level and limiting amino acid supplementation were observed for jejunal crypt depth and ileal villus height and muscle layer thickness at 14 days, as well as for jejunal villus height, V/C ratio, and ileal villus height at 28 days, highlighting the importance of coordinated regulation between protein supply and amino acid balance.

The gastrointestinal tract is not only central to digestion but also represents the largest immune and endocrine organ in the body [14]. Amino acid metabolism is essential for maintaining intestinal integrity and for the synthesis of mucins and immunoglobulins [41]. Methionine, in particular, is a functional essential amino acid involved in gastrointestinal development and antioxidant defense, which is especially important for rapidly growing animals [42]. In this study, balanced methionine supplementation significantly increased total antioxidant capacity (T-AOC) in both the jejunum and ileum of Wanxi white geese at 14 and 28 days of age. Similar protective effects of methionine on intestinal health, antioxidant status, and growth performance have been reported in pigs and poultry [34,43].

Excessively high dietary protein levels may suppress immune function, increase metabolic burden, and impair antioxidant defenses [44,45]. Conversely, in the present study, the 16% CP diet significantly increased malondialdehyde content and catalase activity in the ileum of 14-day-old geese and elevated jejunal catalase activity at 28 days, suggesting that low-protein diets may negatively affect the intestinal antioxidant system during the brooding period. These findings underscore the necessity of balancing limiting amino acids under low-protein conditions to mitigate oxidative stress, preserve intestinal barrier integrity, and maintain redox homeostasis [46,47].

Furthermore, the differential effects of lysine and methionine on intestinal morphology and immune indices may be attributed to the variations in the dietary Lys/Met ratio. In this study, the Lys group maintained a high Lys/Met ratio (approx. 3:1 to 4:1), whereas the Met group had a lower ratio (approx. 2:1). The superior performance of the Met group in antioxidant capacity and late-stage immune organ development suggests that a lower Lys/Met ratio (richer in methionine) might be more beneficial for physiological maintenance and redox balance in goslings, whereas a higher ratio may favor early structural growth of the intestine. Future studies should optimize this ratio dynamically across different brooding stages.

The thymus index is a reliable indicator of immune competence in poultry [48], and proper immune organ development is critical for early-life disease resistance [49,50]. The thymus serves as a central immune organ responsible for T-cell differentiation and maturation, thereby regulating cellular immunity and immune homeostasis [50,51]. Histologically, the thymus consists of clearly defined cortical and medullary regions, and the cortex-to-medulla area ratio reflects thymocyte functional status [52,53,54]. In the present study, while no significant differences were observed at 14 days, balanced methionine supplementation significantly increased the thymus index in 28-day-old geese compared to the lysine-balanced group (*p* < 0.05). This suggests that methionine may play a more prominent role in the later stages of thymic development during the brooding period. Furthermore, no marked alterations were observed in lymphocyte distribution or tissue architecture under different protein levels balanced with lysine or methionine, indicating that amino acid-balanced low-protein diets are sufficient to maintain normal thymic development.

The spleen is a major peripheral immune organ involved in immune regulation, endocrine signaling, and neuroimmune interactions [55,56]. In young geese, splenic immune function is still developing, contributing to their relatively low disease resistance [57,58,59]. In the present study, a significant interaction among dietary protein level, lysine, and methionine was observed for spleen weight at 28 days of age, with methionine supplementation resulting in a relatively higher spleen weight. This suggests a potential enhancement of immune competence during the brooding period. Previous studies have shown that methionine deficiency impairs immune organ development, leading to reduced spleen size and weight in poultry. Collectively, these findings support the critical role of methionine in supporting immune organ development and disease resistance under low-protein dietary conditions.

## 5. Conclusions

This study demonstrates that dietary protein level and the balance of limiting amino acids play critical roles in regulating intestinal development, digestive capacity, antioxidant status, and immune organ growth in Wanxi white geese during the brooding period. Moderate protein reduction, when accompanied by appropriate lysine or methionine supplementation, effectively maintained intestinal structural integrity and functional performance while supporting immune organ development. Among the tested treatments, the 18% crude protein diet achieved the most favorable overall outcomes, suggesting that this level represents an optimal balance between nutritional adequacy and physiological efficiency. These findings provide a theoretical basis for optimizing low-protein feed formulations to improve growth performance, intestinal health, and immune function in Wanxi white geese.

## Figures and Tables

**Table 1:** Experimental design.

	Protein Level	20%	18%	16%
Amino Acid Type				
Lysine (1%)		Group A	Group B	Group C
Methionine (0.5%)		Group D	Group E	Group F

**Table 2:** Composition and nutrient levels of experimental diets (air-dried basis) (%).

Items	Groups					
	A	B	C	D	E	F
Composition						
Corn	52.11	56.89	61.67	52.31	56.81	61.77
Soybean meal	30.00	24.00	18.00	29.60	24.00	18.00
Wheat bran	15.00	16.00	17.00	15.00	16.00	17.00
Limestone	0.75	0.80	0.80	0.75	0.80	0.80
CaHPO_4_	1.36	1.38	1.40	1.36	1.38	1.40
Salt	0.50	0.50	0.50	0.50	0.50	0.50
Mineral premix ^1^	0.05	0.05	0.05	0.05	0.05	0.05
Vitamin Premix ^2^	0.03	0.03	0.03	0.03	0.03	0.03
L-Lys HCL	-	0.15	0.35	-	-	-
DL-Met	-	-	-	0.2	0.23	0.25
Choline Chloride	0.20	0.20	0.20	0.20	0.20	0.20
Total	100	100	100	100	100	100
Nutrient levels ^3^						
Crude protein (CP)	19.94	17.99	16.02	19.93	18.01	15.95
Crude fat (CE)	3.05	3.14	3.21	3.05	3.14	3.25
Metabolizable energy (ME)/(MJ/kg)	11.79	11.79	11.69	11.77	11.78	11.81
Nitrogen efficiency ratio	0.59	0.66	0.73	0.59	0.65	0.74
Crude fiber (CF)	3.62	3.41	3.19	3.60	3.41	3.21
Ca	0.80	0.80	0.79	0.79	0.80	0.79
P	0.76	0.77	0.75	0.77	0.77	0.76
Lysine (Lys)	1.00	0.99	1.00	1.00	0.87	0.73
Methionine (Met)	0.30	0.28	0.25	0.50	0.50	0.50
Threonine (Thr)	0.74	0.65	0.57	0.73	0.65	0.57
Tryptophan (Trp)	0.21	0.20	0.19	0.21	0.20	0.19
Methionine + Cystine (Met + Cys)	0.56	0.57	0.58	0.75	0.86	0.90

Note: ^1^ Mineral premix (per kg of diet): Zn 90 mg, Mn 80 mg, Fe 80 mg, Cu 20 mg, I 0.35 mg; ^2^ Vitamin premix (per kg of diet): VA 9 000 IU, VD3 3 000 IU, VE 24 IU, VB6 3 mg, VK3 1.8 mg, VB1 5 mg, VB2 5 mg, VB12 0.1 mg, nicotinic acid 40 mg, biotin 0.05 mg, D-pantothenic acid 15 mg; the effective content of the supplemented L-Lys HCl was 98.5% and the DL-Met was 99% pure; ^3^ Metabolizable energy were calculated; all other nutrient values were measured.

**Table 3:** Commercial digestive enzyme assay kits used in this study.

Kits	Catalog No.	Supplier
Lipase assay kit	A054-2-1	Nanjing Jiancheng Bioengineer Institute (Nanjing, China)
Trypsin assay kit	A080-2-2	
α-Amylase Assay Kit	C016-1-2	
Maltase assay kit	A082-3-1	
Lactase assay kit	A082-1-1	

**Table 4:** Antioxidant assay kits used in this study.

Kits	Catalog No.	Supplier
Reduced glutathione (GSH) assay kit	A006-2-1	Nanjing Jiancheng Bioengineer Institute (Nanjing, China)
Total antioxidant capacity assay kit	A015-2-1	
Catalase (CAT) assay kit (Ultraviolet)	A007-2-1	
Malondialdehyde (MDA) assay kit	A003-1-2	

**Table 5:** Effects of balanced methionine and lysine diet on the activities of digestive enzymes in the jejunum and ileum mucosa of 14-day-old Wanxi white geese.

Items	Lipase (U/g prot)	Trypsin (U/mg prot)	Lactase (U/mg prot)	Maltase (U/mg prot)	Amylase (U/mg prot)	Lipase (U/g prot)	Trypsin (U/mg prot)	Lactase (U/mg prot)	Maltase (U/mg prot)	Amylase (U/mg prot)
	Jejunum Mucosa					Ileal Mucosa				
A	11.49	93.21	258.87	14.47	0.10	30.52	180.01 ^Bb^	314.58	13.88	0.14
B	12.97	33.56	237.87	14.52	0.11	10.54	128.33 ^Bb^	224.84	10.95	0.11
C	14.84	131.32	279.90	17.77	0.13	8.53	161.77 ^Bb^	291.02	12.24	0.11
D	11.38	75.66	242.51	13.32	0.10	12.78	137.45 ^Bb^	77.25	15.76	0.15
E	11.27	122.09	251.03	10.73	0.12	8.69	272.47 ^Aa^	317.79	19.79	0.18
F	9.18	174.82	276.64	14.98	0.11	9.90	251.90 ^Aa^	177.70	17.20	0.16
SEM	1.43	22.48	33.08	2.02	0.01	8.46	28.89	126.37	1.89	0.02
Amino acid										
Lys	13.09 ^a^	86.03 ^b^	258.88	15.59	0.12	16.53	156.70 ^b^	276.82	12.36 ^Bb^	0.12 ^Bb^
Met	10.61 ^b^	124.20 ^a^	256.73	13.01	0.11	10.46	220.61 ^a^	190.91	17.59 ^Aa^	0.16 ^Aa^
SEM	0.83	12.98	19.10	1.16	0.01	4.89	16.68	72.96	1.09	0.01
Protein level										
20% CP	11.43	84.44 ^Bb^	250.69	13.90	0.10	21.65	158.73	195.92	14.82	0.15
18% CP	12.12	77.85 ^Bb^	244.45	12.63	0.11	9.62	200.40	271.32	15.37	0.15
16% CP	12.01	153.07 ^Aa^	278.27	16.38	0.12	9.22	206.84	234.36	14.72	0.14
SEM	1.01	15.90	23.39	1.43	0.01	5.99	20.43	89.36	1.34	0.01
*p* value										
Amino acid	0.05	0.05	0.94	0.14	0.62	0.39	0.01	0.42	<0.01	<0.01
Protein	0.88	<0.01	0.56	0.20	0.17	0.27	0.22	0.84	0.93	0.69
Amino acid ∗ Protein	0.17	0.09	0.91	0.81	0.25	0.49	0.01	0.44	0.21	0.22

Note: Within the same column, identical or no superscript letters indicate no significant difference (*p* > 0.05), different lowercase letters indicate significant difference (*p* < 0.05), and different uppercase letters indicate extremely significant difference (*p* < 0.01).

**Table 6:** Effects of balanced methionine and lysine diet on the activities of digestive enzymes in the jejunum and ileum mucosa of 28-day-old Wanxi white geese.

Items	Lipase (U/g prot)	Trypsin (U/mg prot)	Lactase (U/mg prot)	Maltase (U/mg prot)	Amylase (U/mg prot)	Lipase (U/g prot)	Trypsin (U/mg prot)	Lactase (U/mg prot)	Maltase (U/mg prot)	Amylase (U/mg prot)
	Jejunum Mucosa					Ileal Mucosa				
A	8.44	112.24	274.23	9.70	0.14	3.75	133.36	347.39	11.91	0.16
B	9.53	185.56	281.34	10.92	0.15	2.94	62.02	235.52	7.15	0.11
C	5.91	135.05	258.43	7.01	0.13	3.47	43.56	238.57	6.26	0.11
D	7.37	121.84	267.11	10.53	0.15	3.01	174.73	299.26	8.36	0.14
E	7.84	81.15	254.25	9.88	0.14	3.71	100.70	298.94	8.51	0.13
F	5.93	81.56	237.41	9.73	0.12	3.84	133.41	293.88	8.00	0.13
SEM	0.90	31.47	18.03	1.31	0.01	0.54	26.94	29.10	1.58	0.01
Amino acid										
Lys	7.96	144.29	271.33	9.21	0.14	3.39	79.65 ^b^	273.83	8.44	0.13
Met	7.05	94.85	252.92	10.05	0.14	3.52	136.28 ^a^	297.36	8.29	0.13
SEM	0.52	18.17	10.41	0.75	0.01	0.31	15.56	16.80	0.91	0.01
Protein level										
20% CP	7.91 ^a^	117.04	270.67	10.12	0.15	3.38	154.04 ^a^	323.33	10.13	0.15
18% CP	8.69 ^a^	133.36	267.79	10.39	0.14	3.32	81.36 ^b^	267.23	7.83	0.12
16% CP	5.92 ^b^	108.31	247.92	8.37	0.13	3.65	88.49 ^b^	266.22	7.13	0.12
SEM	0.63	22.26	12.75	0.92	0.01	0.38	19.05	20.58	1.11	0.01
*p* value										
Amino acid	0.23	0.07	0.23	0.44	0.94	0.76	0.02	0.34	0.91	0.49
Protein	0.02	0.73	0.41	0.27	0.15	0.82	0.03	0.11	0.17	0.08
Amino acid ∗ Protein	0.63	0.22	0.85	0.38	0.63	0.37	0.58	0.13	0.20	0.31

Note: Within the same column, identical or no superscript letters indicate no significant difference (*p* > 0.05), different lowercase letters indicate significant difference (*p* < 0.05).

**Table 7:** Effects of balanced methionine and lysine on relative intestinal length of Wanxi white geese (cm/g).

Items	Jejunum	Ileum	Cecum	Jejunum	Ileum	Cecum
	14-Day-Old			28-Day-Old		
A	73.63 ^b^	7.68	10.46	70.64	9.42	14.80
B	73.25 ^b^	8.37	9.96	70.49	9.87	15.30
C	73.27 ^b^	7.80	9.34	71.20	10.23	16.00
D	73.09 ^b^	7.45	9.34	71.50	9.43	15.50
E	77.12 ^a^	8.12	8.92	69.22	9.30	15.70
F	71.06 ^b^	8.64	10.26	69.37	9.55	14.30
SEM	1.18	0.44	0.48	0.99	0.55	0.67
Amino acid						
Lys	73.38	7.95	9.92	70.78	9.84	15.37
Met	73.76	8.07	9.51	70.03	9.43	15.17
SEM	0.68	0.25	0.28	0.57	0.32	0.38
Protein level						
20% CP	73.36 ^ab^	7.56	9.90	71.07	9.43	15.15
18% CP	75.18 ^a^	8.25	9.44	69.86	9.59	15.50
16% CP	72.16 ^b^	8.22	9.80	70.29	9.89	15.15
SEM	0.84	0.31	0.34	0.70	0.39	0.47
*p* value						
Amino acid	0.70	0.74	0.305	0.36	0.37	0.716
Protein	0.05	0.23	0.611	0.47	0.70	0.833
Amino acid ∗ Protein	0.04	0.38	0.077	0.37	0.80	0.167

Note: Within the same column, identical or no superscript letters indicate no significant difference (*p* > 0.05), different lowercase letters indicate significant difference (*p* < 0.05).

**Table 8:** Effects of balanced methionine and lysine diet on intestinal relative weight of Wanxi white geese (g/g).

Items	Jejunum	Ileum	Cecum	Jejunum	Ileum	Cecum
	14-Day-Old			28-Day-Old		
A	47.62	5.01	5.02	32.74	3.74	4.34
B	57.63	7.03	7.03	35.24	4.14	5.16
C	43.93	5.25	5.25	34.56	4.62	6.03
D	42.61	4.04	4.04	33.73	4.51	4.10
E	41.33	4.21	4.21	35.28	4.42	5.31
F	39.20	4.44	4.44	33.83	4.18	4.33
SEM	4.41	0.88	0.88	1.68	0.39	0.59
Amino acid						
Lys	49.73 ^a^	5.76 ^a^	5.77 ^a^	34.18	4.17	5.18
Met	41.05 ^b^	4.23 ^b^	4.23 ^b^	34.28	4.37	4.58
SEM	2.55	0.51	0.51	0.97	0.22	0.34
Protein level						
20% CP	45.12	4.53	4.53	33.24	4.12	4.22
18% CP	49.48	5.62	5.62	35.26	4.28	5.24
16% CP	41.57	4.85	4.85	34.20	4.40	5.18
SEM	3.12	0.62	0.62	1.19	0.27	0.41
*p* value						
Amino acid	0.02	0.04	0.04	0.94	0.53	0.22
Protein	0.22	0.46	0.46	0.50	0.78	0.17
Amino acid ∗ Protein	0.34	0.46	0.46	0.88	0.31	0.29

Note: Within the same column, identical or no superscript letters indicate no significant difference (*p* > 0.05), different lowercase letters indicate significant difference (*p* < 0.05).

**Table 9:** Structure of the jejunum of Wanxi white geese (μm).

Items	Villus Height	Crypt Depth	Muscularis Thickness	V/C Ratio	Villus Height	Crypt Depth	Muscularis Thickness	V/C Ratio
	14-Day-Old				28-Day-Old			
A	730.33	259.45 ^Aa^	204.34	2.85	1181.34 ^Aa^	275.38	244.82	4.38 ^a^
B	629.76	222.66 ^ABab^	187.85	2.87	1261.35 ^Aa^	312.45	220.38	4.12 ^a^
C	783.34	221.67 b^ABab^	165.35	3.54	873.42 ^Bb^	287.57	183.48	3.03 ^b^
D	668.33	183.46 ^Bb^	183.28	3.72	878.67 ^Bb^	290.62	267.32	3.04 ^b^
E	595.56	187.27 ^Bb^	181.69	3.19	1080.78 ^Aa^	266.67	198.88	4.06 ^a^
F	883.66	273.35 ^Aa^	123.26	3.25	947.38 ^Bb^	257.81	230.27	3.69 ^ab^
SEM	50.31	20.36	11.23	0.34	56.51	17.34	24.42	0.46
Amino acid								
Lys	714.11	234.44	185.56 ^Aa^	3.09	1105.22 ^Aa^	291.66	215.88	3.84
Met	715.66	214.77	162.26 ^Bb^	3.39	968.55 ^Bb^	271.33	231.67	3.60
SEM	29.04	11.75	6.48	0.26	32.63	14.04	14.09	0.27
Protein level								
20% CP	699.33 ^Bb^	221.67 ^ab^	193.83 ^Aa^	3.29	1029.27 ^Bb^	282.83	256.45 ^a^	3.71
18% CP	612.32 ^Bb^	205.16 ^b^	184.25 ^Aa^	3.03	1170.35 ^Aa^	289.23	209.38 ^b^	4.09
16% CP	833.33 ^Aa^	247.66 ^a^	144.50 ^Bb^	3.39	910.68 ^Cc^	272.67	206.62 ^b^	3.37
SEM	35.58	14.39	7.94	0.32	39.96	12.16	17.27	0.33
*p* value								
Amino acid	0.96	0.12	<0.01	0.27	<0.01	0.17	0.28	0.37
Protein	<0.01	0.04	<0.01	0.53	<0.01	0.64	0.02	0.13
Amino acid ∗ Protein	0.09	0.01	0.10	0.25	<0.01	0.23	0.17	0.03

Note: Within the same column, identical or no superscript letters indicate no significant difference (*p* > 0.05), different lowercase letters indicate significant difference (*p* < 0.05), and different uppercase letters indicate extremely significant difference (*p* < 0.01).

**Table 10:** Structure of the ileum of Wanxi white geese (μm).

Items	Villus Height	Crypt Depth	Muscularis Thickness	V/C Ratio	Villus Height	Crypt Depth	Muscularis Thickness	V/C Ratio
	14-Day-Old				28-Day-Old			
A	614.72 ^ABab^	169.46	101.69 ^Bb^	3.66	951.64 ^Bb^	314.35	238.25	3.03
B	566.51 ^Bb^	189.37	131.59 ^Bb^	2.99	1106.28 ^Aa^	343.27	395.54	3.36
C	634.86 ^Aa^	194.28	139.39 ^Bb^	3.29	1128.41 ^Aa^	313.63	230.38	3.64
D	494.62 ^Cc^	183.5	125.28 ^Bb^	2.69	965.19 ^Bb^	204.58	166.38	4.81
E	553.48 ^Bb^	192.49	133.82 ^Bb^	2.94	972.87 ^Bb^	304.28	279.29	3.24
F	535.38 ^Bb^	204.39	227.51 ^Aa^	2.61	1357.53 ^Aa^	301.34	163.72	4.52
SEM	19.04	10.83	20.71	0.22	42.12	24.23	13.28	0.35
Amino acid								
Lys	605.11 ^Aa^	184.86	124.15 ^Bb^	3.31 ^Aa^	1061.78	323.78 ^a^	288.15 ^Aa^	3.34 ^b^
Met	527.56 ^Bb^	193.43	162.48 ^Aa^	2.75 ^Bb^	1098.55	269.61 ^b^	202.39 ^Bb^	4.19 ^a^
SEM	10.99	8.84	11.96	0.17	24.32	13.99	7.66	0.29
Protein level								
20% CP	554.28	176.37	113.25 ^Bb^	3.17	958.39 ^Cc^	259.14 ^b^	202.31 ^Bb^	3.92
18% CP	560.57	190.83	132.73 ^Bb^	2.96	1039.51 ^Bb^	323.37 ^a^	337.15 ^Aa^	3.30
16% CP	584.92	199.51	183.28 ^Aa^	2.95	1242.73 ^Aa^	307.82 ^ab^	196.39 ^Bb^	4.07
SEM	13.46	7.65	14.65	0.15	29.79	24.23	13.27	0.25
*p* value								
Amino acid	<0.01	0.34	0.01	0.01	0.16	0.02	<0.01	0.01
Protein	0.09	0.15	0.01	0.54	<0.01	0.05	<0.01	0.11
Amino acid ∗ Protein	0.01	0.86	0.03	0.14	<0.01	0.16	0.16	0.06

Note: Within the same column, identical or no superscript letters indicate no significant difference (*p* > 0.05), different lowercase letters indicate significant difference (*p* < 0.05), and different uppercase letters indicate extremely significant difference (*p* < 0.01).

**Table 11:** Structure of the cecum of Wanxi white geese (μm).

Items	Villus Height	Crypt Depth	V/C Ratio	Villus Height	Crypt Depth	V/C Ratio
	14-Day-Old			28-Day-Old		
A	235.01 ^Bb^	138.15 ^ab^	1.70 ^ab^	256.82 ^Aa^	148.66 ^Aa^	1.73 ^Bb^
B	191.04 ^Bb^	96.82 ^b^	1.97 ^ab^	209.83 ^Bb^	99.62 ^Bb^	2.58 ^Bb^
C	237.72 ^Bb^	95.73 ^b^	2.18 ^a^	214.14 ^Bb^	106.62 ^Bb^	3.17 ^Aa^
D	183.35 ^Bb^	162.87 ^a^	1.13 ^b^	226.06 ^ABab^	102.95 ^Bb^	2.20 ^Bb^
E	299.13 ^Aa^	160.72 ^a^	1.86 ^ab^	280.86 ^Aa^	105.91 ^Bb^	2.65 ^Bb^
F	215.09 ^Bb^	98.40 ^b^	2.19 ^a^	285.60 ^Aa^	105.77 ^Bb^	2.70 ^Bb^
SEM	5.58	6.79	0.10	5.28	5.16	0.08
Amino acid						
Lys	221.26	110.23	1.95	226.93 ^b^	118.30	2.49
Met	232.52	140.66	1.73	264.17 ^a^	104.88	2.52
SEM	5.75	5.85	0.09	6.12	4.83	0.09
Protein level						
20% CP	209.18 ^b^	150.51 ^ab^	1.42 ^b^	241.44	125.81 ^a^	1.97 ^b^
18% CP	245.09 ^a^	128.77 ^b^	1.92 ^a^	245.35	102.77 ^b^	2.62 ^a^
16% CP	226.41 ^a^	194.13 ^a^	2.19 ^a^	249.87	106.20 ^b^	2.94 ^a^
SEM	5.35	4.13	0.14	4.44	5.50	0.07
*p* value						
Amino acid	0.42	0.23	0.14	0.04	0.21	0.53
Protein	0.04	0.04	0.03	0.33	0.04	0.02
Amino acid ∗ Protein	0.01	0.04	0.02	0.01	0.01	0.01

Note: Within the same column, identical or no superscript letters indicate no significant difference (*p* > 0.05), different lowercase letters indicate significant difference (*p* < 0.05), and different uppercase letters indicate extremely significant difference (*p* < 0.01).

**Table 12:** The antioxidant capacity of the jejunum of Wanxi white goose.

Items	T-AOC (mg/g prot)	GSH (U/mg prot)	MDA (nmol/mg prot)	CAT (U/mg prot)	T-AOC (mg/g prot)	GSH (U/mg prot)	MDA (nmol/mg prot)	CAT (U/mg prot)
	14-Day-Old				28-Day-Old			
A	0.67 ^Bb^	9.59	0.52	2.98	0.74	8.39	0.70	3.59
B	0.83 ^Aa^	6.69	0.67	4.44	0.75	20.94	0.69	3.87
C	0.87 ^Aa^	15.47	0.49	5.51	0.77	19.98	1.27	4.08
D	0.89 ^Aa^	8.40	0.38	3.29	0.88	27.06	0.53	1.80
E	0.91 ^Aa^	9.18	0.20	3.79	0.88	13.82	1.52	4.77
F	0.88 ^Aa^	0.44	0.53	5.57	0.77	16.64	1.95	8.25
SEM	0.04	4.16	0.13	0.99	0.05	7.90	0.31	1.24
Amino acid								
Lys	0.79 ^Bb^	10.59	0.56	4.31	0.75 ^b^	16.44	0.89	3.84
Met	0.89 ^Aa^	6.01	0.37	4.22	0.84 ^a^	19.17	1.33	4.94
SEM	0.02	2.40	0.08	0.57	0.03	4.56	0.18	0.72
Protein level								
20% CP	0.78 ^b^	8.99	0.45	3.13	0.81	17.73	0.61 ^b^	2.70 ^b^
18% CP	0.87 ^a^	7.94	0.44	4.12	0.81	17.38	1.10 ^ab^	4.32 ^ab^
16% CP	0.87 ^a^	7.96	0.51	5.54	0.77	18.31	1.61 ^a^	6.16 a
SEM	0.02	2.94	0.10	0.69	0.03	5.58	0.22	0.88
*p* value								
Amino acid	<0.01	0.19	0.09	0.91	0.03	0.68	0.10	0.29
Protein	0.03	0.96	0.84	0.08	0.64	0.99	0.02	0.04
Amino acid ∗ Protein	0.02	0.11	0.19	0.88	0.26	0.24	0.25	0.08

Note: Within the same column, identical or no superscript letters indicate no significant difference (*p* > 0.05), different lowercase letters indicate significant difference (*p* < 0.05), and different uppercase letters indicate extremely significant difference (*p* < 0.01).

**Table 13:** The antioxidant capacity of the ileum of Wanxi white goose.

Items	T-AOC (mg/g prot)	GSH (U/mg prot)	MDA (nmol/mg prot)	CAT (U/mg prot)	T-AOC (mg/g prot)	GSH (U/mg prot)	MDA (nmol/mg prot)	CAT (U/mg prot)
	14-Day-Old				28-Day-Old			
A	0.74	8.39	0.70	3.59	0.61 ^b^	21.65	1.04	6.81
B	0.75	20.94	0.69	3.87	0.74 ^a^	22.95	1.52	4.07
C	0.77	19.98	1.27	4.08	0.83 ^a^	24.68	1.30	3.88
D	0.88	27.06	0.53	1.80	0.86 ^a^	38.00	0.95	6.24
E	0.88	13.82	1.52	4.77	0.90 ^c^	38.67	0.32	6.41
F	0.77	16.64	1.95	8.25	0.79 ^a^	29.90	0.42	10.11
SEM	0.05	7.90	0.31	1.24	0.04	9.48	0.25	1.56
Amino acid								
Lys	0.75 ^b^	16.44	0.89	3.84	0.73 ^Bb^	23.09	1.29 ^Aa^	4.92
Met	0.84 ^a^	19.17	1.33	4.94	0.85 ^Aa^	35.53	0.56 ^Bb^	7.59
SEM	0.03	4.56	0.18	0.72	0.02	5.47	0.14	0.90
Protein level								
20% CP	0.81	17.73	0.61 ^b^	2.70 ^b^	0.74	29.83	1.00	6.53
18% CP	0.81	17.38	1.10 ^ab^	4.32 ^ab^	0.82	30.81	0.92	5.24
16% CP	0.77	18.31	1.61 a	6.16 ^a^	0.81	27.29	0.86	7.00
SEM	0.03	5.58	0.22	0.88	0.03	6.70	0.18	1.11
*p* value								
Amino acid	0.03	0.68	0.10	0.29	<0.01	0.13	<0.01	0.05
Protein	0.64	0.99	0.02	0.04	0.10	0.93	0.86	0.52
Amino acid ∗ Protein	0.26	0.24	0.25	0.08	<0.01	0.81	0.10	0.12

Note: Within the same column, identical or no superscript letters indicate no significant difference (*p* > 0.05), different lowercase letters indicate significant difference (*p* < 0.05), and different uppercase letters indicate extremely significant difference (*p* < 0.01).

**Table 14:** The antioxidant capacity of the cecum of Wanxi white goose.

Items	T-AOC (mg/g prot)	GSH (U/mg prot)	MDA (nmol/mg prot)	CAT (U/mg prot)	T-AOC (mg/g prot)	GSH (U/mg prot)	MDA (nmol/mg prot)	CAT (U/mg prot)
	14-Day-Old				28-Day-Old			
A	0.21	5.81	0.68	8.76 ^BbCc^	0.53	6.73	0.59	4.30
B	0.22	5.57	0.50	6.68 ^Cc^	0.58	7.05	0.60	9.22
C	0.30	7.19	0.51	11.92 ^AaBb^	0.38	6.51	0.86	9.57
D	0.25	6.71	0.48	12.69 ^Aa^	0.46	6.59	0.37	7.58
E	0.21	7.66	0.55	13.60 ^Aa^	0.34	6.58	0.36	10.09
F	0.21	7.39	0.93	10.39 ^AaBb^	0.47	7.21	0.34	10.62
SEM	0.02	0.54	0.20	1.05	0.10	0.57	0.20	2.19
Amino acid								
Lys	0.24	6.19 ^b^	0.56	9.12 ^Bb^	0.50	6.76	0.68	7.70
Met	0.22	7.25 ^a^	0.66	12.23 ^Aa^	0.43	6.79	0.36	9.43
SEM	0.01	0.31	0.12	0.61	0.06	0.33	0.12	1.26
Protein level								
20% CP	0.23	6.26	0.58	10.73	0.50	6.66	0.48	5.94
18% CP	0.21	6.62	0.52	10.14	0.46	6.82	0.48	9.65
16% CP	0.26	7.29	0.72	11.16	0.43	6.86	0.60	10.09
SEM	0.02	0.38	0.14	0.74	0.07	0.40	0.14	1.55
*p* value								
Amino acid	0.31	0.02	0.59	<0.01	0.37	0.95	0.06	0.34
Protein	0.16	0.17	0.61	0.63	0.77	0.93	0.81	0.14
Amino acid ∗ Protein	0.24	0.23	0.31	<0.01	0.25	0.57	0.70	0.83

Note: Within the same column, identical or no superscript letters indicate no significant difference (*p* > 0.05), different lowercase letters indicate significant difference (*p* < 0.05), and different uppercase letters indicate extremely significant difference (*p* < 0.01).

**Table 15:** Thymus index of Wanxi white goose.

Items	14-Day-Old		28-Day-Old	
	Thymus Weight (g)	Thymus Index (g/kg)	Thymus Weight (g)	Thymus Index (g/kg)
A	1.73	8.01	3.55	3.68
B	2.53	11.82	3.08	3.20
C	1.72	8.47	3.42	3.61
D	2.00	9.95	3.58	3.89
E	1.70	8.43	3.91	4.99
F	1.85	8.91	4.12	4.36
SEM	0.71	3.51	0.94	1.18
Amino acid				
Lys	2.00	9.44	3.35	3.50 ^b^
Met	1.85	9.10	3.87	4.41 ^a^
SEM	0.18	0.93	0.25	0.29
Protein level				
20% CP	1.86	8.98	3.57	3.79
18% CP	2.12	10.13	3.49	4.10
16% CP	1.78	8.69	3.77	3.99
SEM	0.22	1.13	0.30	0.36
*p* value				
Amino acid	0.573	0.798	0.151	0.036
Protein	0.549	0.642	0.804	0.821
Amino acid ∗ Protein	0.183	0.250	0.613	0.295

Note: Within the same column, identical or no superscript letters indicate no significant difference (*p* > 0.05), different lowercase letters indicate significant difference (*p* < 0.05).

**Table 16:** Thymus structure of Wanxi white goose.

Items	14-Day-Old			28-Day-Old		
	Relative Cortical Area (%)	Relative Medullary Area (%)	Cortex-Medulla Ratio	Relative Cortical Area (%)	Relative Medullary Area (%)	Cortex-Medulla Ratio
A	75.37	24.63	3.34	61.20	38.80	1.95
B	61.25	38.75	2.50	74.42	25.58	3.53
C	47.82	52.18	1.61	71.40	28.60	3.33
D	50.71	49.29	1.17	87.40	12.60	6.94
E	50.42	49.58	1.99	74.64	25.36	2.94
F	40.04	59.96	0.69	85.00	15.00	5.79
SEM	0.21	0.21	0.75	0.14	0.14	0.75
Amino acid						
Lys	61.42	38.58	2.48	69.06	30.94	2.94
Met	47.17	52.83	1.28	82.37	17.63	5.23
SEM	0.05	0.05	0.44	0.04	0.04	0.43
Protein level						
20% CP	63.01	36.99	2.25	74.33	25.67	4.45
18% CP	55.84	44.16	2.25	74.62	25.38	3.24
16% CP	43.92	56.08	1.15	78.23	21.77	4.56
SEM	0.06	0.06	0.53	0.08	0.08	0.70
*p* value						
Amino acid	0.066	0.066	0.069	0.128	0.128	0.057
Protein	0.136	0.136	0.291	0.894	0.894	0.507
Amino acid ∗ Protein	0.621	0.621	0.235	0.503	0.503	0.087

**Table 17:** Bursa of Fabricius index of Wanxi white geese.

Items	14-Day-Old		28-Day-Old	
	Bursa Weight (g)	Bursa Index (g/kg)	Bursa Weight (g)	Bursa Index (g/kg)
A	1.32	6.10	0.87	0.89
B	1.58	7.41	0.96	0.98
C	1.22	5.99	1.07	1.14
D	1.10	5.42	1.23	1.34
E	1.24	6.19	1.06	1.33
F	1.13	5.36	1.06	1.12
SEM	0.13	0.69	0.14	0.15
Amino acid				
Lys	1.37	6.50	0.97	1.00 ^b^
Met	1.16	5.66	1.12	1.26 ^a^
SEM	0.08	0.40	0.08	0.08
Protein level				
20% CP	1.21	5.76	1.05	1.11
18% CP	1.41	6.80	1.01	1.15
16% CP	1.17	5.67	1.06	1.13
SEM	0.09	0.49	0.10	0.10
*p* value				
Amino acid	0.058	0.150	0.209	0.039
Protein	0.181	0.216	0.923	0.962
Amino acid ∗ Protein	0.642	0.895	0.413	0.255

Note: Within the same column, identical or no superscript letters indicate no significant difference (*p* > 0.05), different lowercase letters indicate significant difference (*p* < 0.05).

**Table 18:** Bursa of Fabricius structure of Wanxi white geese.

Items	14-Day-Old		28-Day-Old	
	Follicle Area/(μm^2^ × 10^3^)	Medullary Area/(μm^2^ × 10^3^)	Follicle Area/(μm^2^ × 10^3^)	Medullary Area/(μm^2^ × 10^3^)
A	74.46	36.34	221.36	104.75
B	117.26	56.72	254.43	103.78
C	78.91	33.02	199.47	73.07
D	98.66	33.74	289.31	110.27
E	105.23	50.33	232.73	115.27
F	86.64	44.94	176.00	83.77
SEM	7.31	5.73	22.90	13.92
Amino acid				
Lys	90.21	42.03	225.09	93.87
Met	96.84	43.01	232.68	103.10
SEM	4.22	3.31	13.22	8.04
Protein level				
20% CP	86.56 ^Bb^	35.04 ^Bb^	255.33 ^a^	107.51
18% CP	111.25 ^Aa^	53.53 ^Aa^	243.58 ^a^	109.52
16% CP	82.78 ^Bb^	38.98 ^Bb^	187.74 ^b^	78.42
SEM	5.17	4.05	16.19	9.84
*p* value				
Amino acid	0.278	0.836	0.688	0.424
Protein	0.001	0.009	0.016	0.062
Amino acid ∗ Protein	0.065	0.261	0.095	0.973

Note: Within the same column, identical or no superscript letters indicate no significant difference (*p* > 0.05), different lowercase letters indicate significant difference (*p* < 0.05), and different uppercase letters indicate extremely significant difference (*p* < 0.01).

**Table 19:** Spleen index of Wanxi white geese.

Items	14-Day-Old		28-Day-Old	
	Spleen Weight (g)	Spleen Index (g/kg)	Spleen Weight (g)	Spleen Index (g/kg)
A	1.15 ^ab^	5.23	1.30	1.34
B	1.71 ^a^	8.05	1.13	1.17
C	1.44 ^ab^	6.99	1.67	1.80
D	1.59 ^ab^	8.09	1.85	2.04
E	0.99 ^b^	4.98	1.58	2.02
F	1.06 ^b^	5.19	1.62	1.72
SEM	0.18	0.98	0.20	0.26
Amino acid				
Lys	1.44	6.76	1.37	1.44 ^b^
Met	1.22	6.09	1.69	1.93 ^a^
SEM	0.11	0.56	0.11	0.15
Protein level				
20% CP	1.37	6.66	1.58	1.69
18% CP	1.35	6.51	1.36	1.60
16% CP	1.25	6.09	1.65	1.76
SEM	0.13	0.69	0.14	0.18
*p* value				
Amino acid	0.146	0.407	0.059	0.027
Protein	0.788	0.835	0.325	0.818
Amino acid ∗ Protein	0.011	0.054	0.285	0.164

Note: Within the same column, identical or no superscript letters indicate no significant difference (*p* > 0.05), different lowercase letters indicate significant difference (*p* < 0.05).

## Data Availability

The original contributions presented in this study are included in this article, and further inquiries can be directed to the corresponding authors.
